# Visual Influences on Auditory Behavioral, Neural, and Perceptual Processes: A Review

**DOI:** 10.1007/s10162-021-00789-0

**Published:** 2021-05-20

**Authors:** Collins Opoku-Baah, Adriana M. Schoenhaut, Sarah G. Vassall, David A. Tovar, Ramnarayan Ramachandran, Mark T. Wallace

**Affiliations:** 1 Neuroscience Graduate Program, Vanderbilt University, Nashville, TN USA; 2 Vanderbilt Brain Institute, Vanderbilt University, Nashville, TN USA; 3 Department of Psychology, Vanderbilt University, Nashville, TN USA; 4 Department of Hearing and Speech, Vanderbilt University Medical Center, Nashville, TN USA; 5 Vanderbilt Vision Research Center, Nashville, TN USA; 6 Department of Psychiatry and Behavioral Sciences, Vanderbilt University Medical Center, Nashville, TN USA; 7 Department of Pharmacology, Vanderbilt University, Nashville, TN USA

**Keywords:** auditory learning, audiovisual interactions, psychophysical evidence

## Abstract

In a naturalistic environment, auditory cues are often accompanied by information from other senses, which can be redundant with or complementary to the auditory information. Although the multisensory interactions derived from this combination of information and that shape auditory function are seen across all sensory modalities, our greatest body of knowledge to date centers on how vision influences audition. In this review, we attempt to capture the state of our understanding at this point in time regarding this topic. Following a general introduction, the review is divided into 5 sections. In the first section, we review the *psychophysical evidence* in humans regarding vision’s influence in audition, making the distinction between vision’s ability to enhance versus alter auditory performance and perception. Three examples are then described that serve to highlight vision’s ability to modulate auditory processes: spatial ventriloquism, cross-modal dynamic capture, and the McGurk effect. The final part of this section discusses models that have been built based on available psychophysical data and that seek to provide greater mechanistic insights into how vision can impact audition. The second section reviews the extant *neuroimaging and far-field imaging* work on this topic, with a strong emphasis on the roles of feedforward and feedback processes, on imaging insights into the causal nature of audiovisual interactions, and on the limitations of current imaging-based approaches. These limitations point to a greater need for machine-learning-based decoding approaches toward understanding how auditory representations are shaped by vision. The third section reviews the wealth of *neuroanatomical and neurophysiological* data from animal models that highlights audiovisual interactions at the neuronal and circuit level in both subcortical and cortical structures. It also speaks to the functional significance of audiovisual interactions for two critically important facets of auditory perception—scene analysis and communication. The fourth section presents current evidence for alterations in audiovisual processes in three *clinical* conditions: autism, schizophrenia, and sensorineural hearing loss. These changes in audiovisual interactions are postulated to have cascading effects on higher-order domains of dysfunction in these conditions. The final section highlights ongoing work seeking to leverage our knowledge of audiovisual interactions to develop better *remediation approaches* to these sensory-based disorders, founded in concepts of perceptual plasticity in which vision has been shown to have the capacity to facilitate auditory learning.

## INTRODUCTION

We live in a multisensory world, in which we are continually bombarded with sensory information from a variety of sources borne through various forms of environmental energy. Despite the ubiquity of such multisensory information, our knowledge of how information from the different senses is integrated within the brain has lagged behind our knowledge of the processes that support information processing within the individual senses. Fortunately, this is changing as many studies are now probing both the behavioral and perceptual changes that accompany the presentation of stimuli from multiple sensory modalities, as well as the brain mechanisms that support multisensory functions.

From a purely adaptive perspective, having information available from more than a single sense provides tremendous advantages, in terms of both the redundant and complementary information that is conveyed. These benefits have been illustrated in a variety of tasks across almost all possible sensory combinations and have been shown to improve stimulus detection, localization, and response accuracy, as well as to speed responses. In addition, multisensory combinations can often result in categorical shifts in perception, effects that are often best illustrated through illusory phenomena such as the stream-bounce effect, in which the delivery of a sound at the point at which two visual stimuli are streaming through one another gives rise to the compelling illusion of impact and consequent bouncing (Sekuler et al. 1997).

The two best studied sensory systems in regards to multisensory functions are the auditory and visual systems. The reasons for this are many, but interest in these interactions likely stems from the extrapersonal nature of both senses (i.e., they are representing things happening at a distance from the body), the ease with which parametric manipulations of a number of stimulus dimensions can be carried out, and the well-characterized nature of these two senses and their associated brain organization. Within this realm, it seems fair to say that auditory influences on visual function have been more extensively studied (likely a result of the predominance of studies focused on the visual system). Consequently, for the current review, we will focus on the smaller (but rapidly growing) obverse of this, and attempt to provide a comprehensive description of the body of work to-date detailing how the vision can impact auditory function.

The review is divided into five general sections that focus on (1) psychophysical and behavioral studies, (2) neuroimaging, (3) neuroanatomy and neurophysiology, (4) clinical correlates, and (5) learning and rehabilitation.

### Visual Influences on Auditory Perception: Psychophysical Evidence in Humans

As a general rule, we can divide visual influences on auditory perception into two broad categories: perceptual enhancements in which task-relevant or task-irrelevant visual information improves performance on an auditory task, and perceptual alterations, in which conflicting (but task relevant) visual information can change the nature of the auditory percept.

#### Vision Can Enhance Auditory Perceptual Performance

In many circumstances, the stimulation of multiple senses during the performance of a task enhances perceptual and behavioral outcomes (Ernst and Banks 2002; Frassinetti et al. 2002; von Saldern and Noppeney 2013; Zou et al. 2012). In such cases, the brain receives redundant information from the multiple senses about one particular external property. For example, estimating the height of an object using both visual and haptic exploration reduces discrimination thresholds more than using either visual or haptic information alone (Ernst and Banks 2002). Similar perceptual and behavioral enhancements can be observed when task irrelevant or relevant information from one modality affects perceptual judgments specifically related to another modality. In this section, we will review studies demonstrating that visual information can enhance perceptual performance on an auditory task.

#### *Enhancement of Auditory Perceptual Performance by Task-Irrelevant Visual Information*

Task-irrelevant visual information has been shown to have the capacity to enhance perceptual outcomes on a variety of low-level auditory tasks including but not limited to auditory detection (Child and Wendt 1938; Gregg and Brogden 1952; Lovelace et al. 2003), loudness perception (Odgaard et al. 2004), spatial localization (Bolognini et al. 2007), and frequency discrimination (Thorne and Debener 2008). For example, Lovelace et al. (2003) showed that participants’ ability to detect a sound stimulus was enhanced by a task-irrelevant light. In another study, Odgaard et al. (2004) showed that presenting a light together with a white noise increased the perceived loudness of the noise. It has also been shown that vision can play a major role in auditory spatial perception. For example, Bolognini et al. (2007) demonstrated that an auxiliary light can enhance the accuracy of localizing a near-threshold auditory target.

A great deal of the early work focused on multisensory processes was directed toward their low-level sensory features and identified a series of principles closely tied to the statistics of the paired stimuli that played an important role in the resultant interaction seen to a multisensory pairing. Although first described at the level of the single neuron (see Stein and Meredith 1993), these principles were also found to apply to behavioral and psychophysical paradigms. In short, these principles state that the largest multisensory interactions are seen to the pairing of spatially and temporally coincident stimuli, and that the magnitude of the interaction is inversely proportional to the effectiveness of the individual stimuli (see Stein and Meredith 1993). Several studies have shown that the visually induced enhancements observed in auditory perception are constrained by these principles. For example, in Bolognini et al. (2007), an auxiliary light enhanced the accuracy of localizing a near-threshold auditory target more when the light and sound coincided spatially (following the spatial principle) and when the light was less salient (following the inverse-effectiveness principle). In the temporal domain, using an auditory frequency discrimination task, Thorne and Debener (2008) discovered that the most pronounced benefit in response times did not occur when the visual and auditory stimuli were temporally coincident but occurred when the visual stimulus led the auditory stimulus by about 65 ms. Interestingly, while this finding appears to contradict the “temporal principle”, the authors explained that this effect might have resulted from the need for the brain to compensate for the faster processing of audition compared with vision in order to ensure that two signals were temporally aligned when they converged in the brain. In fact, this reasoning is line with studies that have shown that perceptual simultaneity is most often achieved when the visual stimulus leads the auditory stimulus (Zampini et al. 2005a, 2003, 2005b). Together, these studies indicate that perceptual benefits can occur when task-irrelevant visual information is presented together with an auditory stimulus during the performance of auditory tasks, and that the low-level features of these stimuli play an important role in the resultant interaction.

#### *Enhancement of Auditory Perceptual Performance by Task-Relevant Visual Information*

Beyond audiovisual stimuli that share simple spatiotemporal correspondence, perceptual and behavioral benefits can also occur when an auxiliary visual stimulus shares complex, task-relevant features with an auditory stimulus. For example, Møller et al. (2018) showed that presenting a visual stimulus that varied in vertical position—with vertical position known for its correspondence with auditory pitch (Parise et al. 2014, 2016)—facilitated the detection of subtle pitch changes in auditory targets. In another study, Su (2014) showed that a bouncing human point-light figure conveying visual beat information enhanced the ability to perceive and synchronize to auditory rhythms. In both studies, the magnitude of visually induced enhancement of auditory perception was dependent on the level of performance to the auditory stimulus alone. Thus, at the level of both individuals (Møller et al. 2018) and conditions (Su 2014), larger multisensory gains were associated with poorer unisensory auditory performance, consistent with the principle of inverse effectiveness.

Similar visually induced enhancements arising from audiovisual stimulus correspondence are observed in perceptual tasks with more complex and ecologically valid stimuli such as speech. During speech perception, the area of the mouth opening and the acoustic envelope of the speech sound share robust spatial and temporal correspondences (Chandrasekaran et al. 2009). Several studies have demonstrated that being able to visualize the talker’s lip movements significantly enhances comprehension of the auditory speech signal under both good (Arnold and Hill 2001; Reisberg et al. 1987) and noisy listening conditions (Ross et al. 2007; Sumby and Pollack 1954). In addition, visual information from the talker’s mouth movements can aid in the detection of spoken sentences masked by acoustic white noise under noisy conditions (Grant and Seitz 2000). Following the principle of inverse effectiveness, earlier studies on the effect of different levels of noise on the magnitude of visually facilitated speech comprehension and intelligibility reported a monotonic relationship where greater multisensory gains were achieved under very low signal-to-noise conditions (Erber 1969; Sumby and Pollack 1954). However, a recent study by Ross et al. (2007) employing an experimental design that used a relatively larger stimulus set compared with the previous studies demonstrated the maximal multisensory gains were achieved within a range of intermediate signal-to-noise ratios. Unlike the previous studies, the findings from Ross et al. (2007) suggest that there may be a “sweet spot” for multisensory gain at intermediate SNRs. In summary, these findings indicate that task-relevant visual information can enhance perceptual judgments in the auditory domain and that, the degree of enhancement likely depends upon the reliability of the auditory information.

#### *Vision Can Alter Auditory Perception*

Under naturalistic circumstances, auditory and visual information arising from a particular event or object share a number of common features, and, in most cases, integrating them results in perceptual enhancements. However, introducing some degree of conflict between the cues can often result in perceptual transformations best illustrated through several illusions. In this section, we will review three illusions that arise when conflicting visual information is paired with auditory information during the performance of an auditory perceptual task. Together, these illusions have served as a means for understanding how the auditory perceptual system deals with discrepant visual information.

#### *Spatial Ventriloquism*

In the ventriloquist effect, vision has the ability to capture auditory perception when a spatial conflict is introduced between the cues. Historically, the term “ventriloquism” dates back to ancient Greek culture and it literally means “belly talking” (Connor 2000). As a form of entertainment, ventriloquists thrilled their audiences by their ability to synchronize their speech with the lip movements of a puppet while minimizing any movements from their lips. The audience then perceived the speech of the entertainer to emerge from the puppet’s mouth. For a modern demonstration of the ventriloquism illusion, see (https://www.youtube.com/watch?v=yFf5VaYLTNQ&ab_channel=Top10Talent) by Darci Lynne, the winner of America’s Got Talent 2017. Besides being entertaining, the ventriloquist effect also plays a role in our everyday perceptual experiences. For instance, when watching a television at home or at the cinema, we perceive the speech of people talking to originate from their lip movements on the screen although in reality, the speech originates from the speakers positioned elsewhere in the room (Spence and Soto-Faraco 2010). Furthermore, apart from speech, the ventriloquist effect has also been demonstrated using other stimuli such as whistling steaming kettles (Jackson 1953), as well as with simple tones and flashes of light (Alais and Burr 2004; Bertelson and Radeau 1981). Across a number of studies, the ventriloquist effect has been shown to take two general forms: (1) when the perceived location of sound is shifted towards the location of the visual stimulus—so-called cross-modal bias (Alais and Burr 2004; Bertelson 1999; Bertelson and Radeau 1981; Radeau and Bertelson 1987)—and (2) when both the visual and auditory stimuli are perceived at the same location despite substantial spatial disparity—so-called spatial capture (Bertelson and Radeau 1981; Godfroy et al. 2003). Although many of these reports serve to reinforce the dominance of vision over sound in the spatial arena, when the reliability of the visual cue is sufficiently weak, the opposite effect can be seen such that sound seems to attract the visual stimulus (Alais and Burr 2004). This finding implies that the ventriloquist effect is not due to a complete capture of sound by vision but rather as an attempt by the brain to solve the spatial discrepancy by weighting the different cues according to their reliabilities (Alais and Burr 2004).

#### *Crossmodal Dynamic Capture*

In addition to vision modulating the perceived location of static auditory events, visual influences have also been observed for more dynamic auditory stimuli, such as apparent motion. In a series of experiments by Soto-Faraco et al. (2002), participants were presented with both visual and auditory apparent motion stimuli generated by sequentially presenting two flashes of light and two tones. The two speakers and two LEDs (placed each in front of a speaker) were positioned 15 cm to either side of the participant’s midline creating a horizontal apparent motion. Participants were then asked to discriminate the direction of the auditory motion (left or right) while ignoring the visual motion. Unsurprisingly, when the direction of the two stimuli were congruent, participants’ discriminability was near 100% (Soto-Faraco et al. 2002). However, when the direction of visual motion conflicted with that of the auditory motion, discrimination accuracy was reduced by approximately 50%, indicating that the direction of the task-irrelevant visual motion strongly influenced the perceived direction of the auditory motion stimuli (Soto-Faraco et al. 2002). While this finding showed that visual motion information could impact auditory performance, the presence of chance-level performance on the conflict condition indicated that vision may have interfered with the participants’ ability to perceive the auditory motion at all. To test whether this visual influence on auditory motion reflected such interference versus capture, Soto-Faraco and colleagues (2004) in a later study asked participants to perform a similar auditory motion discrimination experiment and to provide confidence ratings after each response. Interestingly, performance under the conflicting motion condition was significantly reduced compared with the near-ceiling performance for the congruent condition even when only trials with highly confident response ratings were included in the analysis. This finding suggests that the observed visual influences were more likely due to visual capture of auditory motion rather than visual interference or guessing. This was due to the fact if the chance-level performance on the conflicting trials reflected guessing, then this effect would be absent for trials where responses were rated highly confident. In addition, and as previously discussed, the ability of vision to influence auditory motion perception was found to be dependent on the spatiotemporal relationship between the auditory and visual stimuli. Thus, the most pronounced effects during the incongruent condition were observed when the two stimuli were presented synchronously and shared the same spatial configuration (Soto-Faraco et al. 2002).

#### *McGurk Effect*

The McGurk effect is a speech-based illusion which occurs when an auditory syllable (phoneme) paired with an incongruent visual syllable (viseme) results in the perception of a novel syllable (MacDonald and McGurk 1978; Mallick et al. 2015; McGurk and MacDonald 1976). In their seminal paper, McGurk and MacDonald (1976) showed that when the phoneme /ba-ba/ was dubbed onto the viseme /ga-ga/, about 80% of preschool children and about 98% of adult observers reported the percept /da-da/. Through subjective experience of the illusion over trials, McGurk and MacDonald (1976) reported that the perception of the McGurk effect was not affected by habituation over time despite the objective awareness of the discrepant nature of the stimuli, indicating its compelling and robust nature. Further highlighting this robustness, the McGurk illusion still occurs in comparable frequency when a point-light image is used to convey visual articulatory information instead of the facial features (Rosenblum and Saldaña 1996) and when the voice and the face of the speaker were gender-mismatched (Green et al. 1991). Due to its robust and simple (i.e., compared with other methods as speech in noise tasks) nature, the McGurk illusion has been used as a tool to index audiovisual integration among children and adults, healthy and clinical populations and furthermore, to study the neural correlates of audiovisual speech perception (see Mallick et al. 2015). However, despite its widespread use, reports of the frequency of perception of the McGurk effect differ dramatically across studies. Using a large sample size (*N* = 360), Mallick et al. (2015) demonstrated that this variability could emanate from differences in the previously used McGurk stimuli, substantial individual differences as well as differences in the experimental procedure used (i.e., open-choice vs forced-choice). To some degree, the individual variability in the susceptibility to the McGurk effect can be attributed to observers’ lip-reading skills (Brown et al. 2018; Strand et al. 2014). Although substantial variability in the frequency of perception of the McGurk effect exists across subjects and stimuli, studies have observed a high test-retest reliability in this illusion, thus indicating its stability within subjects (Mallick et al. 2015; Strand et al. 2014).

#### Mechanistic Principles of Visual Influences on Auditory Perception

Until now, we have provided evidence demonstrating that perceptual and behavioral judgments pertaining to the auditory domain can be influenced by visual information. Specifically, we discussed that auditory perceptual performance can be enhanced when the auditory information is paired with spatially and temporally coincident visual information that is either task irrelevant or relevant. Conversely, pairing visual information that differs from the auditory information in certain stimulus characteristics such as space, time, and semantics often results in the perception of multisensory illusions.

An important question arising from these findings relates to the mechanistic principles governing the observed visual influences on auditory perception. In the case of task-irrelevant visual information enhancing auditory perceptual performance, earlier studies were concerned with whether these enhancements reflected early-stage sensory interactions or late-stage response biases (or both). Indeed, published work provides evidence for both possibilities. For instance, while Odgaard et al. (2004) indicated an early-stage sensory interaction using two complementary experimental manipulations, in support of both mechanisms, Lovelace et al. (2003) found effects of both discriminability and response bias using classic signal detection theory analyses. In fact, the wide differences that have been seen across studies in support of either early sensory or later decisional effects (or both) are likely to be largely a result of differences in task. Collectively, this work seems to strongly suggest that both bottom up (i.e., sensory) and top down influences can ultimately shape the final product of a multisensory interaction. Included in the list of top-down influences that are likely to shape these interactions are cognitive processes such as attention and decisional factors such as task contingencies.

**Fig. 1:**
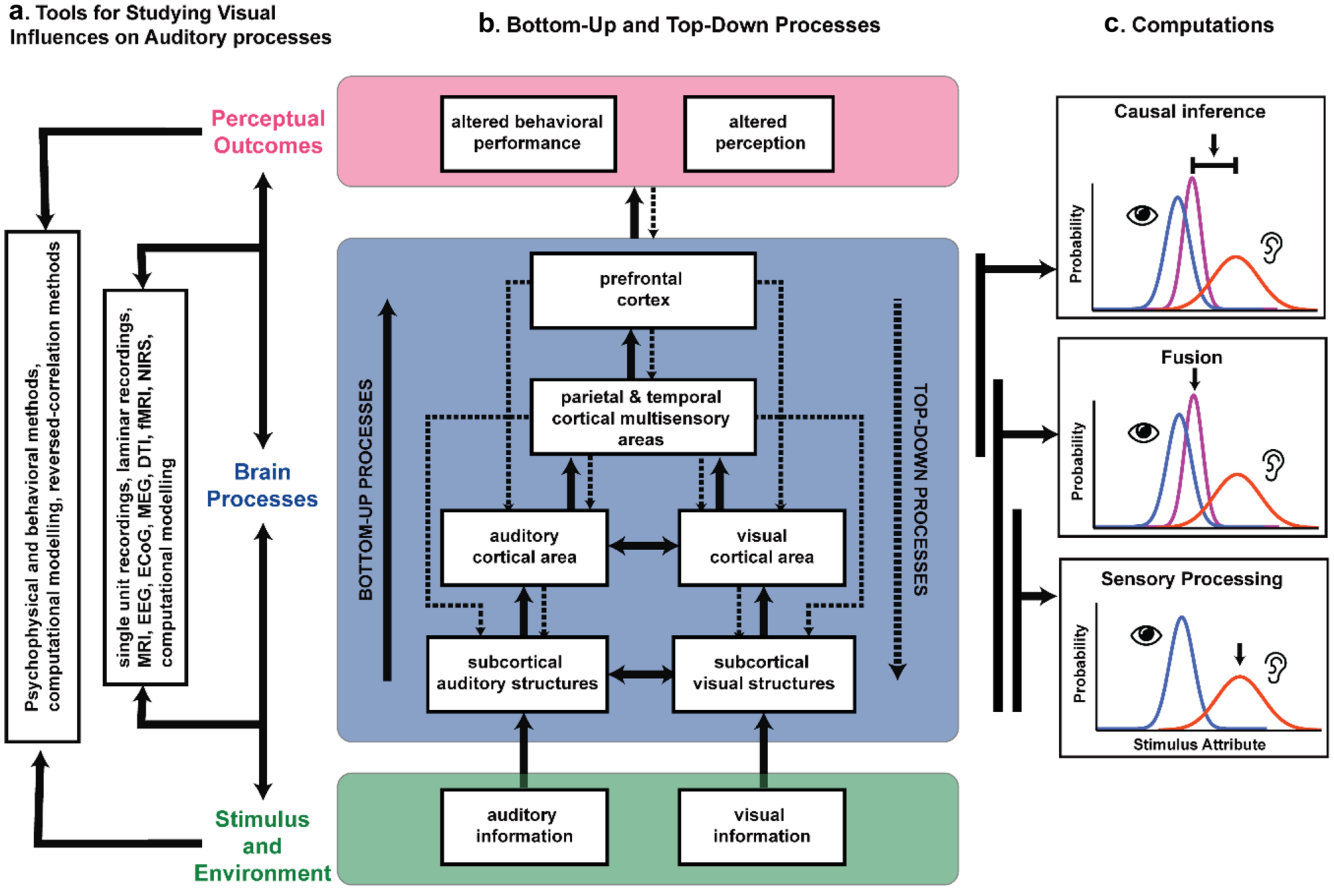
Neural and computational mechanisms of visual influences on auditory processing and perception. **a** Approaches that are used to study how one sensory modality (i.e., vision) influences perception, processing, and plasticity of another sensory modality (i.e., audition). These tools can be divided into those used to study the relationship between stimulus/environmental statistics and perceptual outcomes and those that examine the intervening brain processes that serve to link stimulus and environment to action and perception. **b** Schematic that serves to depict the bottom-up and top-down processes underlying the visual influences on auditory processing and perception, and the associated brain areas. Visual and auditory information (green box) is processed in the brain (middle blue box) along bottom-up (solid arrows) and top-down (dashed arrows) processing streams to produce various behavioral and perceptual outcomes (pink box). **c** Schematic depicting the computations that ultimately underlie the transformation of sensory information into behavioral and perceptual outcomes. Information is initially processed within dedicated sensory areas (bottom panel showing this segregation), then transferred to areas that serve to fuse (integrate) the sensory cues (middle panel), and finally to regions that perform causal inference on the fused stimuli (bottom panel). The extent of the black vertical lines to the left of the boxes indicates the level(s) of the processing hierarchy where these computations are most likely to occur. The boxes in each of the panels display the probability distributions of the auditory (red) and visual (blue) sensory representations and the optimal estimate (shown by black arrow pointing downward; multisensory representation shown in purple) of the stimulus attribute (e.g., location) based on the underlying causal structure. Panel **c** is adapted from Kayser and Shams (2015)

Indeed, as highlighted in Fig. 1, an overarching theme in the body of work surrounding visual influences on auditory processes (and multisensory interactions in general) is the importance of the dialogue between bottom-up (i.e., sensory statistics) and top-down (e.g., attentional, decisional) processes in determining the final product of these interactions. Thus, as highlighted earlier, although low-level stimulus features such as space, time, and effectiveness are key factors in the ultimate product of an audiovisual interaction, these can be strongly modulated (and even overridden) by higher-order features such as task contingencies.

Several studies have suggested that the brain combines sensory signals from multiple modalities relevant to an environmental object or event in order to, first, reach the most reliable (unbiased) estimate and, second, to minimize the variance associated with the final estimate (Ernst and Banks 2002; Ernst and Bülthoff 2004). Importantly, the brain achieves this sensory cue combination, termed maximum likelihood estimation, by weighting the signals according their relative reliabilities (Ernst and Banks 2002; Ernst and Bülthoff 2004). While this model is able to account for circumstances where the signals from the different sensory modalities are spatiotemporally coincident, it appears that when there is moderate or large conflict between the signals, the brain has to decide whether to combine or segregate the signals (Körding et al. 2007). This decision is based on the brain’s ability to infer the unknown underlying causal structure of the signals, which is whether they originate from a common source or different sources (Körding et al. 2007). Based on this, Shams and colleagues developed the causal inference model, which has been applied to several perceptual tasks including spatial localization (Körding et al. 2007; Odegaard and Shams 2016; Odegaard et al. 2017), temporal numerosity (Odegaard and Shams 2016), heading estimation (De Winkel et al. 2017), audiovisual temporal simultaneity judgments (Magnotti et al. 2013); and perceptual phenomena including the spatial ventriloquist effect (Körding et al. 2007; Odegaard and Shams 2016; Odegaard et al. 2017) and the McGurk effect (Magnotti and Beauchamp 2017). Taken together, the evidence suggests that brain employs different strategies when the performance of an auditory task is accompanied by visual information that is either task irrelevant or relevant, or congruent or incongruent. In a highly simplified manner, one can divide the processes required for multisensory interactions into three general steps: the initial processing of the unisensory cues, the fusion of the auditory and visual information, and the ascription of common cause (or not) (Fig. 1). While these models provide conceptual and mechanistic insights into these perceptual changes, neuroimaging experiments in both humans and non-human animals have provided further understanding into the neural correlates and signatures of these visual influences on auditory perception. We take a deeper look into these areas in subsequent sections.

### Neuroimaging and Far-Field Recording: Neural Evidence for Cross-modal Modulation of Auditory Processes

To understand the neural underpinnings of how other sensory modalities can influence auditory processes, whether they are task relevant or task irrelevant, first requires a broader look into the functional organization of the brain. The neocortex has been commonly segmented into areas dedicated to processing incoming information from our five senses (Felleman and Van Essen 1991). However, this compartmentalization has been questioned by many studies (Kayser et al. 2005, 2008; Martuzzi et al. 2007; Murray et al. 2005) leading some to the other extreme—is the entirety of neocortex multisensory (Ghazanfar and Schroeder 2006)? Indeed, a number of fMRI studies in blind individuals have shown that in the absence of vision, visual cortex activation commonly associated with visual objects is utilized to encode sound objects (Amedi et al. 2003; van den Hurk et al. 2017; Vetter et al. 2014). Similar recruitment of auditory areas and reweighting of visual cues has been found in deaf individuals and cochlear implant users (Benetti et al. 2017; Bola et al. 2017; Butera et al. 2018). Overall, these studies demonstrate the brain’s capacity for marked cross-modal plasticity, in which areas normally associated with one sensory modality can be influenced (and even taken over) by other sensory modalities. Further, they speak to a general ability of the brain to use information across senses to optimize encoding of information even at early sensory areas.

#### *Evidence of Feedforward Cross-modal Modulation of Auditory Processes*

Conceptually, there are two broad ways by which multisensory processes might modulate audition—during the feedforward pass of auditory processing or through convergence in association cortices following the initial feedforward sweep and subsequent feedback (Brandman et al. 2020). Activation early along the auditory hierarchy by other sensory modalities suggesting potential feedforward modulation has been found in a number of studies. For instance, an fMRI optogenetic study in rats found that excitation of infragranular excitatory pyramidal neurons in V1 enhanced auditory subcortical BOLD responses in the inferior colliculus (Leong et al. 2018).

At the level of the cerebral cortex, an fMRI study found that noise bursts activated primary visual cortex and checkerboards activated primary auditory cortex, and when presented together, these stimuli shortened the latency of the hemodynamic BOLD response in each area, indicating multisensory facilitation (Martuzzi et al. 2007). In another fMRI study, investigators showed movies consisting of video, audio, and audiovisual components to awake and anesthetized macaques. Here, they found that core and belt auditory cortical areas were activated by just the visual components of the movie and demonstrated audiovisual convergence in the caudal portion of primary auditory cortex, as well as in belt and parabelt areas (Kayser et al. 2008). Similarly, touch has been shown to modulate activity in early auditory areas with integration of touch and sound in the auditory caudal belt (Kayser et al. 2005). Using EEG, combined somatosensory and auditory stimulation has been found to elicit multisensory responses greater than the summed responses of either sound or touch alone as early as 50 ms post-stimulus onset (Murray et al. 2005).

A number of different mechanisms may underlie these modulations of feedforward auditory processes. One potential mechanism is through oscillatory phase resets across the different sensory modalities (Fries 2015). Links between phase reset and perception were found in an electrocorticography (ECoG) study in which epilepsy patients performed a speeded reaction time test in which they were asked to identify the presence of visual, auditory, and audiovisual stimuli. In the audiovisual condition, it was found visual stimulation modulated auditory activity via phase reset in delta and theta bands. Furthermore, stronger synchrony between regions led to faster reaction times (Mercier et al. 2015). Similar phase resets have also been noted in a number of other studies (Romei et al. 2012; Simon and Wallace 2017). However, it is important to note that oscillations can also play a role through attentional mechanisms with phase resets coming through feedback from supramodal areas (Lakatos et al. 2009). Further mechanisms by which other sensory modalities might influence auditory processes include nonspecific increases in membrane potential. They may come from increased arousal or other mechanisms such as stochastic resonance—the phenomenon where inserting noise into a non-linear system such as the human brain paradoxically increases perceptual awareness (Fujioka et al. 2006; Lugo et al. 2008). Interestingly, these mechanisms propose an avenue by which feedforward modulation of auditory processes does not require a casual structure between sensory modalities.

#### *Evidence of Feedback Cross-modal Modulation of Auditory Processes*

Top-down enhancement from feedback processes do rely on higher level semantic properties to help with causal inference, helping bind sensory stimuli that are coming from a common source (Körding et al. 2007). Speech in particular relies on binding the semantic components found in visual and auditory streams. In a study using EEG and fMRI, subjects listened/viewed auditory and visual syllables alone, congruent audiovisual syllables, and incongruent syllables. It was found that the reliability of the visual component increased connectivity between visual and auditory cortices, but the congruence (or lack thereof) of the audiovisual stimulus increased (or decreased) the connectivity between superior temporal sulcus (STS) and primary visual and auditory areas (Arnal et al. 2009). Further MEG and EEG studies found that there was a shift in oscillations from delta oscillations (3–4 Hz) in congruent speech to beta high-gamma coupling (15 Hz, 60–80 Hz) in incongruent and noisy speech in STS (Arnal et al. 2011; Schepers et al. 2013). A recent EEG study has further found that delta oscillations (1–4 Hz) specifically track speech comprehension, whereas theta (4–8 Hz) tracks speech clarity (Etard and Reichenbach 2019). To further investigate the role of vision on speech comprehension, one study manipulated the timing between visual and auditory stimuli. In this study, it was found that perception was better when audio lagged behind video and resulted in reduced activity in STG, presumably due to inhibition of phonemes that would not be compatible with the video (Karas et al. 2019). These results complement another study which manipulated subjects’ expectations of upcoming words, showing priming effects in STG at about 100 ms latency (Wang et al. 2019). Together, these results point to the importance of the STG in speech perception.

Using the McGurk illusion described previously in this review, researchers have further established the role of STG and STS in auditory and visual integration. Specifically, left STS responses have been shown to predict McGurk illusion on a subject-by-subject basis with stronger STS activity related to a stronger illusion (Nath and Beauchamp 2012). Furthermore, transcranial magnetic stimulation of STS from 100 ms prior to audio onset to 100 ms following auditory onset led to disruption of McGurk illusion (Beauchamp et al. 2010). Studies have speculated that one role the STS may be performing is resolving incongruence between visual and auditory components in a top-down manner through observed late beta oscillations (Roa Romero et al. 2015).

While top-down modulation occurs in association cortices, such as the STG, top-down influences can extend as far back as primary auditory cortex. A recent MEG study showed that visual lip reading can create a coarse auditory speech representation in early auditory cortices, independent of initial auditory input (Bourguignon et al. 2020). Complementing this finding, a study found frequency-specific neural patterns from auditory predictions that activated auditory cortex in a tonotopic fashion (Demarchi et al. 2019). The interplay between feedforward and feedback processes was delineated further in a 7 T fMRI study where subjects viewed visual, auditory, and audiovisual stimuli with varying levels of attention. Remarkably, they found that audiovisual interactions were found most prominently in infragranular layers of primary auditory cortex whereas attentional influences were most evident in the supragranular layers, suggesting distinct circuits for these processes (Gau et al. 2020).

#### *Causal Structure and Cross-modal Modulation*

The interplay between feedforward and feedback activity has led to further exploration of the role of causal inference in multisensory integration. Recent EEG studies have suggested that multisensory integration occurs in a hierarchical manner beginning with an initial segregation of information at the level of the early sensory cortices, followed by information fusion according to stimulus reliability in intermediate areas, and finally by causal inference in decision-making areas which ultimately determines whether the stimuli should remain fused or segregated (Cao et al. 2019; Rohe and Noppeney 2018) (see Fig. 1). However, what defines an early area, intermediate area, and area needed for decision making? And is this gradient fixed or can it change depending on how relevant the multisensory information is to behavior? These are important questions as even within the STG, demarcations have been found between anterior and posterior STG with decisional activity localizing to more posterior regions (Ozker et al. 2017). The demarcation is corroborated by studies that show anterior STG responds more vigorously to clear auditory components while posterior STG responds more vigorously when speech has lower signal to noise, suggesting that posterior STG is more sensitive to the reliability of the incoming visual and auditory signals and thus more suited to perform for multisensory integration (Ozker et al. 2017). Further supporting this idea, a study which measured correspondence between layers of a convolutional neural networks trained to classify between different music genres and human fMRI found that anterior STG has more correspondence with low level auditory features and posterior STG showing greater correspondence with deeper layers optimized to classify/decide between music genres (Güçlü et al. 2016).

#### *Limitations of Neuroimaging Studies and Striving Towards Solutions*

In this section, we have reviewed a number of fMRI and EEG studies that have provided insights into where and when auditory information is modified by information from other sensory modalities. However, it is important to note that these methods are far removed from the underlying neural spikes and the traditional neurophysiological methods used to assess multisensory integration. The fMRI signal is a hemodynamic signal that has been shown to diverge from neural spiking under conditions of perceptual suppression (Maier et al. 2008; Self et al. 2019). In these circumstances, it more closely resembles the low-frequency local field potential. Local field potentials in turn form the basis of EEG and MEG studies, which provide an estimation of the synaptic inputs in a given location. However, LFP signals carry potential problems when investigating multisensory integration, as they are known to volume conduct across electrodes (Kajikawa and Schroeder 2011). Thus, applying the multisensory concepts of superadditivity (Wallace et al. 1998) at a given electrode becomes difficult as it can simply reflect the activity of a neighboring brain structure without necessarily signifying that there is integration (Laurienti et al. 2005).

There are a number of ways to address these issues. One is to use decoding methods to abstract the amount of information present in a multisensory signal when compared with unisensory signals (Jung et al. 2018; Tovar et al. 2020). Here, the added activation will only be beneficial if it carries added unique information from each sensory modality. Additionally, a decoding framework makes it possible to fuse the information gained from EEG and fMRI and place them into a common computational space with the use of representational similarity analysis (Cichy and Pantazis 2017; Cichy et al. 2014, 2016; Kriegeskorte et al. 2008). While these techniques will certainly help better assess multisensory integration and cross-modal modulation, it does not remedy all potential issues. Critically, animal studies, which can link neural spiking to LFP, and further connect these measures to behavior, are necessary for a better grasp of how auditory processes can be modified by vision and other sensory modalities.

### Neuroanatomical and Electrophysiological Evidence of Visual Influences on Auditory Processes in Animal Models

#### *Methods to Study Audiovisual Interactions in the Auditory System*

Multisensory integration at a single neuron level refers to the significant difference in a neuron’s firing rate or discharge pattern evoked in response to a multisensory stimulus compared with that evoked by those stimuli presented individually (Stein and Meredith 1993). As highlighted above, single neuron studies were the basis for the spatial, temporal, and inverse effectiveness principles. Indeed, electrophysiological recordings in animal models provide a powerful method to investigate visual influences on auditory processing due the ability to directly measure activity from single neurons as well as neural populations, sometimes even in the presence of simultaneous behavior (Logothetis 2008). Complementing these physiological studies has been a host of anatomical tracing studies, which allow for the mapping of visual inputs onto auditory structures, and thus the basis for the functional interactions that are seen.

#### *Visual Inputs and Audiovisual Responses in Subcortical Regions*

The first structure along the auditory pathway to show input from visual structures is the inferior colliculus (IC), with the majority of these inputs targeting extralemniscal regions of the IC (Cooper and Young 1976). The IC is an essential auditory structure, with almost all ascending auditory information processed here before being transmitted to the thalamus (Aitkin and Phillips 1984). Anatomical studies in rodents (hamsters, guinea pigs, and mole-lemmings) and non-human primates (NHP) have shown retinal innervation of the pericentral nucleus of the IC (ICP) (Herbin et al. 1994; Itaya and Van Hoesen 1982; Yamauchi and Yamadori 1982) and the external nucleus of the inferior colliculus (ICX) (Cooper and Cowey 1990). Additionally, both the central nucleus of the inferior colliculus (ICC) and the ICX receive input from the visual cortex (Cooper and Young 1976). The primary source of visual information in the IC, however, comes from the superior colliculus (SC), a multisensory midbrain nucleus implicated in the control of gaze (Wallace et al. 1998), with reciprocal connections to several regions of IC (Stitt et al. 2015).

Given these visual inputs into the IC, it is no surprise that in cat, owl, and NHP models, IC neurons respond to visual stimuli both in the presence and absence of concurrent auditory stimuli (Bergan and Knudsen 2009; Gutfreund et al. 2002; Mascetti and Strozzi 1988; Porter et al. 2007; Tawil et al. 1983). In NHPs, both excitatory and inhibitory responses to visual stimuli were seen, although excitatory responses were approximately six times more prevalent (Porter et al. 2007). Visually responsive neurons were most common in regions of the IC without specific frequency tuning (Bulkin and Groh 2012), presumably the dorsal cortex and the external nucleus. It is important to note that the Bulkin and Groh study found a larger proportion of visually responsive neurons than previous studies, likely a result of the fact that the animals were engaged in a task (Mascetti and Strozzi 1988; Tawil et al. 1983). Furthermore, in concordance with the temporal and spatial principles of multisensory integration, the visual modulation of single unit auditory responses in the owl ICX have been shown to be dependent on the spatial and temporal correspondence of the stimuli (Bergan and Knudsen 2009).

#### Hypothesized Function of Audiovisual Interactions in the Inferior Colliculus

It has been suggested by several studies in owls that audiovisual interactions within the IC function to help calibrate a map of auditory space, even though visual stimuli in isolation have little effect on neuronal activity (Bergan and Knudsen 2009; Brainard and Knudsen 1993; DeBello et al. 2001; Gutfreund et al. 2002). Evidence for this has come from experiments in which the visual field is displaced by prismatic spectacles, inducing a recalibration of the auditory space map to align with the new visual map in the optic tectum, the equivalent of the mammalian SC (Knudsen and Brainard 1991a). Further supporting this hypothesis is the finding that auditory responses in the IC of NHPs are modulated by shifts in gaze direction, a behavior that would require a representation of eye position within auditory space to accurately localize an auditory target (Groh et al. 2001).

More recent studies in mice have suggested that visual inputs to the IC serve to increase the sensitivity of auditory information processing (Cheng et al. 2019). This study found that auditory frequency responses in ICC were sharpened or flattened in a frequency-specific manner in the presence of visual stimuli (light flashes) under free-field conditions (Cheng et al. 2019). The sharpening of tuning curves may allow for finer frequency analysis at a wider range of frequencies, whereas the flattening of these curves may function to stabilize responsiveness to specific frequency bands. A similar experiment looking at visual modulation of auditory responses as a function of sound pressure level in IC revealed sound pressure level-specific enhancement or suppression of activity (Cheng et al. 2020). Visual modulation was strongest for stimuli presented at a given neuron’s preferred auditory azimuth. Thus, while it is unlikely that visual modulation plays an essential role in the formation of an auditory space map in the mammalian IC, it might have the ability to modulate this map by increasing sound level sensitivity (Cheng et al. 2020).

#### *Visual Inputs and Audiovisual Responses in Auditory Cortex*

The exact sources of visual input to certain areas of the auditory cortex that lead to visually modulated auditory responses are still widely unknown; however, recent studies have begun to reveal some of these connections. Visual input likely comes from “multisensory” nuclei of the thalamus, such as suprageniculate limitans and the magnocellular division of the medial geniculate complex (de la Mothe et al. 2006) as well as from association and limbic cortical regions often thought of as multisensory, such as the superior temporal polysensory area (STP), area 7a of the parietal cortex, medial parietal areas 23/31, and the claustrum (Smiley and Falchier 2009). Whereas the visual inputs from thalamus are almost certainly feedforward in nature, those from association cortices are likely feedback in nature, a suggestion supported by laminar analyses in monkey auditory cortex (Schroeder and Foxe 2002).

Although far from comprehensive, some studies have implemented tracer injections and retrograde labeling to look at projections from visual to auditory cortices. In one such study conducted in ferret, Bizley et al. (2007) were able to identify visual input to core and non-core auditory areas from ipsilateral visual areas 17, 18, 19, 20 and in which the non-core regions of auditory cortex were more densely innervated. In addition, they were able to identify area 20 of visual cortex as the largest source of visual input to the core of auditory cortex (Bizley et al. 2007). In gerbils, tracing studies have shown projections from Oc2, the second visual area, to auditory cortex (Budinger et al 2006). In macaque, Falchier et al. (2010) revealed projections from areas V2 and prostriata of the visual cortex to parts of belt, parabelt, and temporoparietal area (Tpt). In contrast, however, Cappe and Barone (2005) did not find connections between V2 and prostriata to the core of marmoset auditory cortex. Collectively, these results show evidence for visual inputs into auditory cortex, but also suggest significant species differences in the connectivity patterns.

The effects of visual signals on auditory processing can be seen using neurophysiological approaches in both the core and extralemniscal belt regions of auditory cortex. In macaque, 12% of auditory core neurons were found to show exhibit audiovisual interactions, which typically manifested as response suppression (Kayser et al. 2009). In cat, single units in the cortex of the anterior ectosylvian sulcus (AES)—a non-core auditory cortical region—exhibit both frank audiovisual responsiveness as well as visual modulation of auditory responses (Clarey and Irvine 1986; Meredith and Allman 2009; Wallace et al. 1992, 1993). In both ferret and macaque, single units in core and non-core regions of auditory cortex have also been shown to respond to a visual stimulus presented alone (Bizley et al. 2007; Kayser et al. 2008; Leinonen et al. 1980). Some of these visually responsive neurons (4% in macaque) also responded to auditory stimuli presented alone, therefore classifying them as bimodal neurons (Kayser et al. 2009). While single unit responses to visual stimuli were present across stimulus conditions and in different regions of the auditory cortex, it is important to note that the fraction of neurons exhibiting these properties is far less than the fraction of recording sites showing audiovisual interactions in LFPs.

Work in macaque auditory cortex illustrates that audiovisual interactions seen here depend on low-level stimulus characteristics such as timing and effectiveness, suggesting some degree of universality to the principles of multisensory integration (Kayser et al. 2010, 2008). In addition to showing the characteristic changes in firing rate resulting from multisensory stimulation, studies in this model have highlighted that visual influences on auditory processing also extend to benefits in trial-by-trial reliability (Kayser et al. 2010).

#### *Functional Significance of Audiovisual Integration in Auditory Cortex*

#### *Auditory Scene Analysis*

A central aspect of auditory processing is the perceptual segregation of competing sound sources. This phenomenon was initially described as the cocktail party problem (Cherry 1953) and has since been described in terms of auditory scene analysis, or sound source segregation. For many years, such naturalistic scenes were rarely modeled in neurophysiological studies. However, a recent study by Atilgan et al. (2018) in which LFPs were recorded from the auditory cortex in behaving ferrets suggests integration of auditory and visual information may support auditory scene analysis by capitalizing on the temporal coherence of auditory and visual signals. Crucially, this integration was found to result in the enhanced encoding of an orthogonal sound feature, timbre, representing a critical piece of evidence toward the formation of an auditory object. The finding that temporal coherence supports auditory scene analysis has been supported by a number of other studies as well. Namely, investigators have consistently reported that cortical oscillations play a role in processing competing sound sources (e.g., competing speech streams) and stimuli from multiple modalities (Lakatos et al. 2007; Zion Golumbic et al. 2013). Specifically, in the case of visual modulation of auditory processing, it has recently been shown that visual input (a rhythmic flashing LED) enhances oscillations entrained by sound in primary auditory cortex (O'Connell et al. 2020). This enhanced excitability may reflect a mechanism for prioritized processing of temporally coherent auditory and visual stimuli. While these mechanisms which may subserve auditory scene analysis have largely been explored in auditory cortex, it is highly likely that they are in play in other brain areas as well. One example of this was seen in a study in the cochlear nucleus and which found that neurometric functions based on single-unit activity paralleled stream segregation behavior in humans (Pressnitzer et al. 2008). Another is seen in non-lemniscal regions of the inferior colliculus, where the presence of prediction error signaling (as evidenced in an oddball paradigm) may play an important role in sound source segregation (Parras et al. 2017; Valdés-Baizabal et al. 2020).

#### *Processing of Communication Signals*

As described in previous sections, imaging and behavioral studies have long emphasized the advantage of having information from multiple sensory channels for optimal communication in humans (and even non-human primates). Facial gestures are particularly useful for listeners during speech perception. Behaviorally, visible mouth movements have been shown to improve vocalization detection in macaque monkeys (Chandrasekaran et al. 2013). Electrophysiology studies in macaques have begun to reveal the neural correlates of such audiovisual benefits in the communication realm. In one such study, Ghazanfar et al. (2005) found that pairing macaque vocalization sounds with a video of the animal vocalizing altered LFPs in both core and lateral belt regions of the macaque auditory cortex (Ghazanfar 2009; Ghazanfar et al. 2005). However, this modulation was not seen when the video was a moving disk simulating facial movements. Similar naturalistic stimuli pairings were used by Kayser et al. (2010) while recording activity from single units in core and belt auditory regions of the macaque, and these pairings resulted in increases in firing rate and spike pattern reliability across trials (Kayser et al. 2010). More recently, it has been shown that the addition of conspecific visual stimuli leads to improved processing of communication signals via shorter latency responses in auditory cortical neurons (Chandrasekaran et al. 2013). While the exact mechanisms behind how auditory communication signal processing is enhanced with the addition of visual information in macaque auditory cortex is still under active investigation, it is likely that this enhancement is at least partly dependent on changes in oscillatory dynamics (Ghazanfar et al. 2008).

### Clinical Models of Atypical Visual Influence on Auditory Processing and Cognition

In addition to invasive animal studies, human studies of clinical populations are also useful for illuminating the mechanisms underlying visual modulation of audition. The advent of the National Institute of Mental Health’s Research Domain Criteria (RDoC) has led to a significant paradigm shift in psychiatric research in the last few years. The RDoC framework aims to elucidate the physiological mechanisms underlying phenotypic traits across disorders rather than analyzing symptomology of disorders in isolation. A medical model of psychiatric disorders makes it possible to identify common physiological aberrations across disorders, assess physiological effects on symptomology, and compare psychiatric disorders to other non-psychiatric disorders and diseases. Using a similar, literature-based approach, this section aims to reveal some of the structural and functional abnormalities across three distinct clinical populations: autism spectrum disorder (ASD), schizophrenia (SZ), and sensorineural hearing loss (SNHL), with an emphasis on how auditory function is influenced by vision.

Additionally, functional and structural changes relevant to both unisensory (i.e., auditory alone) and multisensory processing will be discussed. Of particular interest is the posterior regions of temporal cortex, the structure and function of which have been shown to be altered in all three clinical groups. The posterior superior temporal sulcus (pSTS) has been identified as a critical node for assessing audiovisual relationships (Wallace and Stevenson 2014), as well as for processing social information such as facial features (Hotier et al. 2017; Isik et al. 2017) and lexical-semantic processing (Friederici et al. 2009). Stronger within-network connectivity in the right pSTS and resting state functional connectivity between the right pSTS and right occipital face area, early visual cortex, and bilateral STS are correlated with better facial expression recognition abilities (Wang et al. 2014). The posterior superior temporal gyrus (pSTG) is associated with phonological and semantic information processing (Chang et al. 2015) as well as speech perception in noisy environments (Ozker et al. 2017). Core symptoms of ASD, SZ, and SNHL include altered sensory processing and atypical social communication, and it is therefore likely that the abnormalities observed in these regions of temporal cortex are correlated with these changes in perceptual processes.

#### *Autism Spectrum Disorder*

A core symptom of ASD is abnormal sensory experiences, including hypersensitivity, hyposensitivity, and sensory seeking (American Psychiatric Association 2013). A striking dichotomy has been seen in autism as it relates to benefit and decrements in sensory processing. For example, in audition, evidence has accumulated for intact or even superior low-level processing abilities (e.g., pitch perception), but impaired performance in more complex tasks (Baum Miller and Wallace 2019). Additionally, there is evidence for deficits in auditory pattern formation, which manifests as reduced response amplitudes to novel speech and non-speech auditory stimuli (Baum Miller and Wallace 2019; Giraud et al. 2001; Lodhia et al. 2014; Seery et al. 2014). This dichotomy does not appear to be unique to the auditory system, as children with ASD show comparable neural and behavioral signatures in discriminating first-order (e.g., luminance-defined) visual stimuli, but perform worse than TD controls in discriminating second-order (e.g., texture-defined) stimuli (Rivest et al. 2013). Children with ASD have also been shown to trend toward local processing requirements in embedded figure (Manjaly et al. 2007) and visual construction tasks (Kim et al. 2020). These abnormalities in auditory and visual pattern formation are likely to play a key contributory role to the well-established difficulties in higher-order cognitive processes that accompany autism, such as the core symptom of impaired social communication.

Indeed, prior research has demonstrated impaired speech perception in noisy environments in ASD (Baum Miller and Wallace 2019; Haigh et al. 2016; Stevenson et al. 2018). Performance in speech-in-noise tasks, as described in previous sections, is enhanced when synchronous, congruent visual stimuli are paired with auditory stimuli. However, the same benefit is not experienced in many individuals with ASD, and structural and functional abnormalities in the temporal areas described above may underlie this decreased performance. Additionally, atypical functional connectivity of the pSTS (Wallace and Stevenson 2014) has been observed in the ASD population. Given the importance of the pSTS in multisensory processing and extraction of higher-order auditory and visual features, it is likely that these physiological differences map to behavioral expression of decreased social attention.

For example, infants later diagnosed with ASD show increased fixation toward the mouth as opposed to eyes or other social features (Jones and Klin 2013), and an overall increased fixation time and saccades directed at non-social images compared with social images (Pierce et al. 2016). Additionally, adults with ASD have been shown to gaze less at eyes, with no significant group differences in mouth- or total face-directed gaze (Dalton et al. 2005; Fujioka et al. 2016; Spezio et al. 2007), and these gaze differences are correlated with decreased performance in face emotion recognition compared with TD individuals (Baron-Cohen 2017). Structural abnormalities of the pSTS are correlated with this deficit. It was found that the right anterior caudal ramus of the pSTS is longer in some patients with ASD, and the length of this structure is negatively correlated with fixation time to eyes and performance on emotional recognition tasks (Hotier et al. 2017). Additionally, individuals with ASD display difficulty extracting affective prosody from auditory speech (Brooks et al. 2018). Interestingly, Tardif et al. (2007) found that unlike TD children, ASD children’s categorization of emotional facial expressions was facilitated by congruent vocalizations as well as slower presentation of stimuli, which may suggest decreased salience of visual social stimuli, and a resulting deficit in synthesizing higher order auditory and visual information into a unified perceptual representation. Furthermore, prior research appears to illustrate an impairment of visual signals in supplementing the auditory representations, perhaps due to the atypical auditory or visual representations and/or atypical integration of the unimodal stimuli.

More insight can also be gleaned from examining cortical oscillations and the role of STS in regulating excitation/inhibition. Balz et al. (2016) demonstrated that the concentration of the inhibitory neurotransmitter GABA in the STS was significantly correlated with gamma-band oscillation power. This research suggests that a proper balance of excitation/inhibition is critical for regulating cortical oscillations, which facilitate feature binding by synchronizing activity within and across different cortical regions (Stone et al. 2014). An increased excitatory/inhibitory (E/I) ratio (either by increased excitation or decreased inhibition) has been theorized in models of ASD (Foss-Feig et al. 2017; Seymour et al. 2019), and research has demonstrated increased baseline gamma activity as well. Aberrant E/I ratios and oscillatory power in ASD therefore appear to be in part responsible for impaired feature binding within and across sensory modalities. Results from studies of the McGurk illusion demonstrate that children with ASD are less likely to perceive the McGurk illusion (Baum Miller and Wallace 2019; Meilleur et al. 2020), perhaps as a function of atypical gamma-band oscillations and an inability to synchronize cortical activity. Additionally, individuals with ASD perform worse on tasks that require attention to biological motion (Blake et al. 2003; Swettenham et al. 2013) and social information (Baum Miller and Wallace 2019), which would suggest impaired ability to fuse auditory and visual cues at high levels of complexity.

Finally, general cortical organization should be noted when considering ASD symptomology. There is an apparent lack of left hemispheric lateralization for language in ASD individuals (Baum Miller and Wallace 2019), which likely contributes to language impairments in the population. Left hemispheric lateralization is known to be implicated in language processing, and prior research has linked a lack of left lateralization in core language regions (including the STG) with language impairments (de Guibert et al. 2011). In summary, individuals with ASD appear to present with atypicalities in higher-order unimodal auditory processing, which result in impaired grouping and response to novel stimuli. Impaired auditory processing, along with weaknesses in visual and audiovisual processes, are likely to have cascading effects that ultimately give rise to the more clinically recognized changes in social communication.

#### *Schizophrenia*

Researchers and clinicians often compare symptomology and underlying physiology of schizophrenia (SZ) to that of ASD—despite several critical differences in presentation. Autism and SZ do share common symptoms, such as abnormal sensory experiences (e.g., auditory hallucinations in SZ; (American Psychiatric Association 2013) and impaired social communication (American Psychiatric Association 2013). Additionally, individuals with SZ also exhibit impaired temporal auditory discrimination (Stevenson et al. 2017), impaired auditory oddball detection (Cook et al. 2012), reduced electrophysiological responses to novel stimuli (Jahshan et al. 2013), impaired speech perception in noisy environments (Haigh et al. 2016; Stevenson et al. 2017), and lack of left lateralization for language (Ocklenburg et al. 2013).

Evidence also suggests changes in pSTS in those with SZ. Abnormal pSTS activity is prevalent in individuals with SZ, including hyperactivity during neutral face processing and hypoactivity during emotion recognition when compared with neurotypical (NT) counterparts (Mier et al. 2017). This atypical activity also extends to the broader face area, which has been correlated with decreased influence of emotional faces on emotional voice categorization (Liu et al. 2016). Similar to ASD symptoms, deficits in extraction of prosodic information are seen in SZ groups (Jahshan et al. 2013). In this instance, atypical visual influence on communication appears to be more prominent among higher-order feature extraction and integration. Similar to ASD, individuals with SZ appear to experience significant difficulty synthesizing higher-order features of the social environment into a unified representation, which may be a result of impaired audiovisual integration in conjunction with atypical unimodal auditory processing.

Taken together, this evidence suggests that atypical activity in the temporal cortex is correlated with impaired ability to extract and integrate high level sensory information, such as emotional prosody, which leads to decreased ability to form contextual representations of naturalistic speech. Further illustrating the role of the pSTS, the function of which is correlated with regulation of E/I as described above, an increased E/I ratio has been theorized in models of SZ (Foss-Feig et al. 2017; Stevenson et al. 2017), and research has demonstrated increased baseline gamma activity and altered stimulus-evoked gamma activity (Foss-Feig et al. 2017).

Individuals with SZ show an impaired ability to perceive the McGurk illusion, which de Gelder et al. (2003) found to be correlated with impaired lipreading and an auditory bias toward the AV stimuli. Collectively, the picture of sensory function in SZ parallels that seen in ASD, with documented impairment in unimodal auditory processing coupled with challenges in the ability to properly integrate meaningfully paired visual stimuli with their appropriate auditory counterparts. One major consequence of these changes is their impact on higher-order social communication.

#### *Sensorineural Hearing Loss and Cochlear Implant Users*

Individuals with sensorineural hearing loss (SNHL) experience difficulties in temporal auditory discrimination, both for low-level features (Dincer D’Alessandro et al. 2018), including temporal fine structure (needed for pitch perception for prosody) (Zeng 2002), as well as for speech perception (Liberman 2017). Individuals with SNHL also appear to show impairments in higher-order auditory grouping, despite an increased reliance on lipreading during AV speech (Giraud et al. 2001; Huyse et al. 2013). For example, children with SNHL show deficits in 2-talker babble tasks (Goldsworthy and Markle 2019), and adults with hearing impairment exhibit reduced cortical suppression of distractors (Dai et al. 2018). An informative study using the chinchilla model of hearing loss demonstrated distorted tonotopic coding of temporal fine structure and envelope (Henry et al. 2016), which may contribute to the impaired signal detection in noise and parcellation of relevant auditory signals observed in SNHL patients.

For the SNHL population, a wide array of differences have been noted in temporal cortex. Li et al. (2012) demonstrated decreased regional synchronization of the middle STS (mSTS) in people with acquired deafness (AD), as well as weaker connectivity between mSTS and anterior STS in congenitally deaf individuals and AD individuals compared with controls. This connectivity was found to be correlated with overall language impairment. Unilateral hearing loss has also been correlated with decreased grey matter in the left temporal gyrus (Yang et al. 2014), which may directly contribute to poor speech-in-noise perception given the role of this region in speech comprehension.

Individuals with uncorrected SNHL experience unique auditory and audiovisual challenges, particularly in speech tasks, some of which are shared by individuals with cochlear implant (CI)-corrected SNHL. For example, the impaired ability to group and discriminate auditory signals coupled with the poor speech-in-noise intelligibility in CI users is likely a function of impairments in selective attention and/or auditory grouping. Prosodic extraction from speech is also problematic for CI users, although it is likely that the lower-level acoustic features that are lost, such as place of articulation (Giraud et al. 2001), might be better explained by the design of CI speech processors and the direct stimulation of the cochlear nerve (Naples and Ruckenstein 2019).

Changes in cortex extend beyond auditory regions in hearing loss. For example, in post-lingually deaf CI users, Giraud et al. (2001) found that the early visual cortex (V1/V2) was employed when participants listened to native speech with their eyes closed. It is believed that this effect is caused by the experience-dependent adaptations that rely heavily on visual input, such as lipreading during AV speech (Giraud et al. 2001; Huyse et al. 2013). Interestingly, adult-deafened rats have been shown to exhibit reduced temporal sensitivity in the multisensory zone of lateral extrastriate visual cortex (V2L) after deafening, caused by increased preference for visual stimulation; in contrast, the auditory zone of V2L has been shown to become more responsive to visual stimuli and able to effectively process AV stimuli comparably to the pre-deafened multisensory zone in AV processing tasks (Schormans and Allman 2019; Schormans, Typlt, and Allman 2017). Thus, both animal and human models suggest that reorganization favors visual inputs in the absence or reduction of auditory input.

In contrast to observed effects in the McGurk illusion among individuals with ASD and SZ, Huyse et al. (2013) found that children with CI performed comparably on a McGurk task compared with their normal hearing counterparts, although non-proficient CI users provided significantly more visual-based answers when the illusion was not perceived. These results suggest a greater reliance on visual cues during speech perception, which complements the above findings that V1/V2 is employed in CI users during auditory-only listening. Reductions in auditory-evoked gamma responses as a consequence of an altered E/I ratio have also been shown in individuals with hearing loss and are correlated with reduced response suppression and impaired speech-in-noise performance compared with individuals with normal hearing (Ross et al. 2020).

Finally, recent research by Lazard and Giraud (2017) found that a subset of CI users displayed a lack of left lateralization for phonological processing, which correlated with faster response times in speech tasks, but associated with poorer overall CI outcomes. Although this appears counterintuitive, faster response times may not equate to increased accuracy, and it is likely that the above-described abnormalities in auditory processing contribute to impaired AV speech processing at numerous levels.

### The Role of Vision in Auditory Learning: the Future of Rehabilitation

The complex interplay that occurs between the senses not only impacts on-going processing and perception, but also likely plays an important role in future processes through its impact on learning and brain plasticity. Currently, several studies in the area of perceptual learning have provided evidence that indicates that training paradigms that employ information from multiple senses yield better perceptual outcomes using fewer sessions than training paradigms using information from a single modality (Kim et al. 2008; Opoku-Baah et al. 2020; Seitz et al. 2006; Shams and Seitz 2008). Indeed, these findings support the idea that rehabilitation based on audiovisual training may be the future of therapeutic interventions for individuals with certain type of perceptual deficits. For this review, we discuss how vision influences learning in auditory spatial perception and in auditory speech perception and communication. Furthermore, we highlight studies that show that incorporating vision into these auditory perceptual training paradigms may improve outcomes in patients with ASD, SZ, hearing loss, and cochlear implantations.

#### *Visual Facilitation of Auditory Spatial Learning*

The ability to locate auditory events precisely and rapidly is advantageous for the survival of many species, whether it is to identify suitable mating partners, search for food or prey, or escape from predators (King 2009). In contrast to the visual system where spatial information is mapped directly onto the retina, the auditory system uses computational processes to infer the location of auditory events from acoustical cues that arise from complex interactions between sound waves and structures such as the head and the external ears (Recanzone and Sutter 2008). As a result, the auditory system possesses poorer spatial resolution relative to the visual system (Bruns and Röder 2019). While localizing auditory events can be accomplished using only auditory cues, several studies have demonstrated that accompanying visual information can influence this process (King 2009). As discussed in earlier sections, when sound sources are accompanied by spatially and temporally coincident visual information, auditory localization accuracy tends to improve (Bolognini et al. 2007; Shelton and Searle 1980). Conversely, when meaningful disparity exists between the physical locations of the auditory and visual stimuli, auditory positional judgments tend to shift towards the location of the visual stimulus (i.e., spatial ventriloquism) (Alais and Burr 2004; Bertelson 1999; Bertelson and Radeau 1981). Whether the auditory and visual cues share similar or different spatial locations, it has been suggested that, when sufficiently close, the brain combines both sensory cues in order to enhance the reliability of its spatial estimate (Alais and Burr 2004).

It has been well established that the mechanisms involved in the representation of auditory space can undergo changes during and beyond the sensitive periods of development (King 2009). Importantly, several studies have highlighted the dominant role vision plays in this auditory spatial plasticity. During development when plasticity is at its peak, vision is known to guide the maturation of the spatial response properties of auditory neurons and ensure proper alignment of intersensory spatial maps in the superior colliculus (King et al. 1988; Knudsen and Brainard 1991b; Wallace and Stein 2007). Furthermore, the effect of vision on inducing long-term changes in spatial localization behavior (Brainard and Knudsen 1998) and the structure of auditory spatial maps is observed in studies using older animals (Bergan et al. 2005; Brainard and Knudsen 1998; Knudse 1998). In humans, rapid changes in auditory spatial localization are seen following a brief passive exposure to temporally coincident but spatially discrepant audiovisual signals. After such an exposure, observers typically mislocalize the sound stimulus toward the location of the visual stimulus even in the absence of visual stimulation. This phenomenon, termed the ventriloquism aftereffect, reflects rapid recalibration of auditory space (for reviews, see Chen and Vroomen 2013; Recanzone 2009) and has also been observed in non-human primates (Kopčo et al. 2009; Woods and Recanzone 2004).

While presenting spatially conflicting audiovisual signals can induce crossmodal spatial recalibration, training on a spatial localization task using spatially and temporally congruent audiovisual signals results in subsequent improvement in auditory spatial localization performance in the absence of the visual stimulus (Berger et al. 2018; Cai et al. 2018; Passamonti et al. 2009). In addition, these performance enhancements observed after the audiovisual training paradigms can transfer to untrained spatial locations, indicating the generalizability of these training effects (Cai et al. 2018). Although the implementation of the audiovisual training paradigms differs across these studies, together, these results promise an effective utilization of audiovisual spatial localization training paradigms for recalibrating altered spatial processing and perception in individuals with hearing loss and cochlear implantation. To corroborate the effectiveness of these paradigms for rehabilitating individuals with deficits in auditory spatial localization, Isaiah et al. (2014) showed that training bilaterally cochlear implanted adult ferrets with early-onset hearing loss improved their ability to localize sound in the horizontal plane. Interestingly, visual facilitation of auditory spatial localization training can occur when training is implemented in virtual reality headsets (Berger et al. 2018). The portable and somewhat convenient nature of these headsets make implementing home-based training paradigms more feasible and thus, beneficial to patients who cannot access in-person laboratory-based training due to old age, limited mobility and/or long commute times. In addition, audiovisual training paradigms have been shown to improve localization performance under monaural conditions (Strelnikov et al. 2011; Zonooz and Van Opstal 2019), where spatial localization abilities are severely hampered (Colletti et al. 1988; Luntz et al. 2005; Nava et al. 2009; Slattery and Middlebrooks 1994). Taken together, these studies provide evidence that highlights the crucial role of vision in auditory spatial learning and plasticity and further indicate that audiovisual perceptual learning paradigms can be effective tools in improving auditory perceptual outcomes especially for spatial localization in cochlear implant individuals and in individuals with hearing loss.

#### *Visual Facilitation of Auditory Learning in Speech Perception and Social Communication*

It is clear from studying neurotypical individuals that utilizing training methods that couple meaningful, complementary auditory and visual stimuli can drastically improve performance on speech identification and learning. Indeed, speech is inherently multimodal, and redundant visual and auditory information provides salient cues about the speaker and speech content (von Kriegstein and Giraud 2006). Redundancy in the form of congruent, synchronous AV stimuli has been shown to elicit faster recognition (von Kriegstein and Giraud 2006), as well as more robust identification (Sheffert and Olson 2004; von Kriegstein and Giraud 2006) and learning (Schall et al. 2013; Zäske et al. 2015) of voice identity. The effect of AV training, compared with auditory only training, is magnified when participants are asked to identify speaker identity (Zäske et al. 2015) or recall words (Sheffert and Olson 2004) spoken by familiar voices compared with unfamiliar voices. Additionally, in speech-in-noise tasks, it has been observed that individuals trained with AV stimuli significantly surpass performance of those trained with auditory-only stimuli (Lidestam et al. 2014).

Interestingly, this effect is also observed in secondary language learning, and may provide valuable insight into the mechanisms by which language learning is facilitated by visual input. A study by Hazan et al. (2005) found that Japanese learners of English showed similar rates of improvement in discrimination of certain syllables across training conditions, but pronunciation of these syllables was significantly improved in individuals who received naturalistic AV training compared with those who received AV training with a synthetic face or auditory-only training. The results of this study suggest that visual training facilitates speech production and intelligibility, and it is likely that this effect is due to increased visual information, coupled with the equivalent formants of auditory speech.

The results of the combined research suggest that there is significant facilitation of auditory comprehension and learning in the presence of salient visual cues, as well as an improvement in speech production. Indeed, imaging studies have demonstrated that greater connectivity between voice and face areas (von Kriegstein et al. 2008; von Kriegstein and Giraud 2006) as well as faster stimulus-evoked activity in the fusiform face area (Schall et al. 2013) following AV face-voice training. Therefore, novel therapeutic interventions geared toward individuals with ASD, SZ, and/or SNHL would benefit from integrating similar evidence-based practices into current standard of care. As of now, there is some promising research suggesting the efficacy of such novel therapies. These treatments have the potential to improve critical areas of concern related to speech comprehension, as well as higher-level AV tasks such as emotion recognition and social attention.

In addition, AV models of speech, with specific emphasis on speech intelligibility, have shown significant promise in improving speech comprehension. For example, a three-dimensional (3D) audiovisual visualization model of human faces, which provides virtual representations of speech as well as models of the mouth and internal articulatory movements (e.g., air flow), have shown promise in improving speech intelligibility in ASD (Chen et al. 2019). Additionally, an AV training paradigm for speech recognition has shown improvement in speech intelligibility among hearing aid and CI users (Sato et al. 2020). Complex skills such as face emotion recognition may also be enhanced in ASD after training using a 3D feedback game (White et al. 2018). The aim of each of these novel interventions is to provide the user with redundant auditory and visual information in order to augment higher-order processes such as emotion recognition.

In addition to three-dimensional models, AV training to recognize speech in noise has shown to be particularly effective for individuals with ASD and SNHL. One paradigm that presented naturalistic speech with increasing noise in the presence of a synchronous speaking face resulted in participants being better able to identify untrained speech in noise (Irwin et al. 2015). Similarly, elderly adults with compensated hearing loss showed significant improvement in speech-in-noise identification when trained with AV models, whereas auditory-only training had no effect (Moradi et al. 2017). Most importantly, Moradi et al. (2017) reported training effects that persisted for a month after the close of the training period.

#### *Mechanisms Underlying the Visual Facilitation of Auditory Perceptual Learning*

Whether the task-relevant sensory modality is audition or vision, several studies including those presented above have demonstrated that perceptual learning protocols that engage multiple senses provide better learning outcomes than those that involve information from only the task-relevant sensory modality. These findings raise important questions about the mechanistic principles underlying the more pronounced learning benefits after multisensory training and furthermore, how these principles could aid the development of novel therapies for the management of clinical conditions such as SNHL, ASD, or SZ.

Shams and Seitz (2008) suggested that multisensory training that facilitates unisensory learning may alter how information is represented in unisensory structures in two ways. First, it is possible that signals from brain areas of the auxiliary modality can modulate neuronal activity in the brain area of the task-relevant modality leading to enhanced plasticity and changes in the neuronal properties of these neurons over the course of learning. Consequently, presenting unisensory stimuli after training will strongly activate the unisensory structures leading to enhanced perception. Another possibility is that the presence of information from the auxiliary sensory modality during training can lead to enhanced connectivity between the unisensory areas, or altered processing in the multisensory structures. Here, both mechanisms are likely to lead to enhanced perception stemming from an activation of a wider network of brain areas during subsequent presentation of unisensory stimuli.

Indeed, while these two mechanisms—changes in unisensory structures and changes in multisensory structures—may co-occur to facilitate learning, many of the studies that have investigated the neural mechanisms underlying multisensory-facilitated learning have shown evidence for the latter. For example, Zilber et al. (2014) demonstrated that a short period of audiovisual training on motion discrimination recruited a wider network of brain areas (including pSTS, mSTS, and AC), which was activated above baseline during the post-training phase when the auditory stimulus was absent. Additionally, it has been shown that AV training using both face and voice information resulted in an increase in functional connectivity between the face (i.e., fusiform face areas) and voice (i.e., temporal voice areas) brain areas (von Kriegstein et al. 2008; von Kriegstein and Giraud 2006). Interestingly, the finding of increased connectivity between brain areas involved in multisensory perceptual learning has also been demonstrated using a temporal simultaneity judgment task (Powers et al. 2012).

In conclusion, there seems a consensus that when multiple senses are engaged in learning, the brain areas of the senses involved are recruited and the connectivity patterns between them are strengthened, leading to an activation of a wider network during subsequent processing of unisensory information and improved perception. In fact, other perspectives suggest that the recruitment of supramodal brain areas during multisensory perceptual learning may enhance the generalizability of learning, thereby extending the benefits of learning to other stimuli, activities, or locations (Proulx et al. 2014).

## Concluding Remarks

As should be clear from the above, we have learned a great deal about how auditory behavioral, neural, and perceptual processes can be influenced by vision, and more generally about the ubiquity and utility of multisensory interactions in shaping our actions and perceptions. The general theme of this work is that under naturalistic circumstances we are almost continually challenged with information coming from multiple senses, and that the brain makes use of both redundant and complementary information in order to generate adaptive behavioral benefits and to create a coherent perceptual reality. Given this, it is not terribly surprising that the brain combines and integrates information across the senses, and that this convergence and consequent integration takes place at a number of processing stages, including those once thought dedicated to unisensory (i.e., auditory alone) function.

Although much has been learned about visual influences on auditory processes, a number of unanswered questions and areas of future inquiry remain. Advances in neurophysiological methods have allowed the opportunity to record from large neuronal ensembles across multiple brain areas simultaneously in animal models, and will undoubtedly provide critical insights into sensory encoding and information transfer. Such approaches make feasible the ability to see the transformations of information that characterize moving up scales of analysis and computation (e.g., from individual neurons to local circuits). In addition, they enable these transforms to be captured as one moves from node to node within the processing hierarchy and as information moves from sensory representation to decision. Such a capacity will also allow greater insight into the dialogue between “bottom up” and “top down” processes that ultimately shape these computations. As alluded to previously, application of machine learning to carry out sophisticated pattern analyses on both neurophysiological and imaging datasets has already provided significant insight into the nature of neural representations, most notably when these representations are sparse and highly distributed. Continued efforts in this area should significantly add to our knowledge of how auditory representations are influenced by vision. In keeping with the computational theme, the continued development of biologically plausible models such as those that attempt to instantiate Bayesian causal inference processes will also shed great light on these questions. Finally, and as should be clear from the final sections, ongoing efforts need to better elucidate how audiovisual function is altered in clinical contexts, and how such alterations relate to core domains of dysfunction. For, with such knowledge in hand, we can begin to build and apply remediation approaches that are founded in improving sensory function (and multisensory integration), and that have the potential for having cascading effects into higher-order cognitive and executive function domains.
